# Insights into virulence: structure classification of the *Vibrio parahaemolyticus* RIMD mobilome

**DOI:** 10.1128/msystems.00796-23

**Published:** 2023-11-28

**Authors:** Lisa N. Kinch, R. Dustin Schaeffer, Jing Zhang, Qian Cong, Kim Orth, Nick Grishin

**Affiliations:** 1 Department of Molecular Biology, University of Texas Southwestern Medical Center, Dallas, Texas, USA; 2 Howard Hughes Medical Institute, University of Texas Southwestern Medical Center, Dallas, Texas, USA; 3 Department of Biophysics, University of Texas Southwestern Medical Center, Dallas, Texas, USA; 4 Eugene McDermott Center for Human Growth and Development, University of Texas Southwestern Medical Center, Dallas, Texas, USA; 5 Harold C. Simmons Comprehensive Cancer Center, University of Texas Southwestern Medical Center, Dallas, Texas, USA; 6 Department of Biochemistry, University of Texas Southwestern Medical Center, Dallas, Texas, USA; Pacific Northwest National Laboratory, Richland, Washington, USA

**Keywords:** protein structure-function, genome analysis, comparative studies, mobile genetic elements, virulence, pathogenicity islands, food-borne pathogens, *Vibrio parahaemolyticus*, evolution

## Abstract

*Vibrio parahaemolyticus* (*Vpar*) is a major cause of seafood-related gastroenteritis. A pandemic strain, RIMD2210633 (RIMD), has emerged from ancestors due to virulence mechanisms acquired through horizontal gene transfer (HGT). Mobile genetic elements play an essential role in RIMD pathogenicity, but they evolve rapidly and are challenging for sequence-based homology detection and functional inference. Guided by the Evolutionary Classification of protein Domains (ECOD), we utilized structure-assisted homology detection of high-quality AlphaFold models for RIMD proteins to augment their functional interpretation. This approach detected 7,107 domains from 3,970 RIMD proteins and classified them into the ECOD hierarchy, providing functional insights into 80% of the proteome. RIMD proteins originating from HGT were identified by comparison against 86 complete proteomes of other *Vpar* strains. The resulting RIMD mobilome is enriched with phage markers and bacterial defense domains. Evolutionary classification of known HGT proteins suggests they provide selective advantages against the human host, phage, and other stresses. Compared to a pre-pandemic strain, we found fast-evolving proteins with potential involvement in RIMD virulence and phage avoidance. These findings provide vital insights into bacterial evolution, pathogenic mechanisms, and adaptation strategies while offering testable hypotheses for experimental studies and an online resource for future investigation of the RIMD proteome.

**IMPORTANCE:**

The pandemic *Vpar* strain RIMD causes seafood-borne illness worldwide. Previous comparative genomic studies have revealed pathogenicity islands in RIMD that contribute to the success of the strain in infection. However, not all virulence determinants have been identified, and many of the proteins encoded in known pathogenicity islands are of unknown function. Based on the EOCD database, we used evolution-based classification of structure models for the RIMD proteome to improve our functional understanding of virulence determinants acquired by the pandemic strain. We further identify and classify previously unknown mobile protein domains as well as fast evolving residue positions in structure models that contribute to virulence and adaptation with respect to a pre-pandemic strain. Our work highlights key contributions of phage in mediating seafood born illness, suggesting this strain balances its avoidance of phage predators with its successful colonization of human hosts.

## INTRODUCTION

*Vibrio parahaemolyticus* (*Vpar*), a primary causative agent of gastroenteritis from raw seafood consumption, naturally inhabits marine and estuarine waters (1, 2). The bacterium has adapted different lifestyles to survive in these aquatic environments and their host organisms (3, 4). While most isolates of *Vpar* are non-pathogenic, a pandemic O3:K6 serovar isolate, RIMD2210633 (RIMD), has emerged with multiple acquired virulence mechanisms, and the incidence of disease outbreaks is increasing with rising seawater temperatures (5, 6). Genome comparisons of RIMD with other *Vpar* strains revealed considerable genomic exchange within this species and the acquisition of multiple genomic islands in the virulent RIMD strain (7–15). These regions encode a type III secretion system (T3SS2) that injects a unique set of effector proteins into host cells to establish an intracellular replicative niche and two type VI secretion systems (T6SS1 and T6SS2) that inject effectors into competing bacterial cells to enhance RIMD’s fitness in marine environments (3, 16, 17). Yet, less is known about the function of other islands.

The term “pan-genome” was coined to represent the entire set of genes belonging to multiple strains of a microbial species. The pan-genome includes a core set of genes shared by all strains and an accessory genome of partially shared and strain-specific genes (18). Analysis of the pan-genome from the family Vibrionaceae has revealed both the extent of horizontal gene transfer (HGT) among strains and species (up to 75% of total genes in the family Vibrionaceae) as well as how genetic variability influences pathogenicity and niche adaptation (19). These easily exchanged mobile genetic elements (MGEs) can influence the composition of microbial communities and contribute to environmental selection pressures (20). Many computational tools exist to streamline the study of MGEs (21, 22). However, given their frequent roles in virulence and host interactions, sequences acquired by HGT are often subject to fast evolution that compromises functional and phylogenetic interpretations (23–25).

Identifying homologs of fast-evolving proteins using their amino acid sequences has been proven difficult, even for the most sensitive search methods (26–28). Protein structures are more conserved than sequences (29–31). Thus, determining structures of fast-evolving virulence proteins and identifying their relationship to known folds have traditionally informed function (32–34). Recent progress in protein structure prediction has shown that deep-learning networks such as AlphaFold can achieve atomic-resolution results, even for fast-evolving proteins (35–40). Folds rendered by these structure prediction methods can provide insight into distant homologs that have lost their sequence signals. For example, VtrA and VtrC encoded by the RIMD T3SS2 pathogenicity island serve as a co-component signal transduction system that turns on transcription of the T3SS2 virulence machine and its effectors in response to host bile acid. This system could only be identified in other enteric bacteria using their common domain and operon organization combined with structure comparison of AlphaFold models (41).

Recently, AlphaFold protein structure DataBase (AFDB) (42) has released models for over 200 million proteins, including those encoded by the RIMD genome. Such predictions should provide an invaluable resource for the functional annotation of RIMD proteins, especially for those that have evolved beyond sequence detection limits. One method for analyzing large numbers of predicted protein structures is to place them in context with an evolution-based database of domains from experimental structures. The Evolutionary Classification of Domains (ECOD) database provides such a resource, which has been used in combination with an automated Domain Parser for AlphaFold Models (DPAM) to classify the human proteome (33, 43, 44). ECOD is a hierarchical classification of protein domains from experimentally determined structures in Protein Data Bank (PDB), and domains are partitioned into groups by overall architecture (A-group), homology (H-group), and similar topology (T-group). A similar genome-wide classification of RIMD models into the ECOD hierarchy should expand our knowledge of pathogenic adaptations in RIMD MGEs that remain largely unknown.

This study provides an evolution-based classification of AlphaFold models for the RIMD proteins. The proteome was parsed into 7,696 domains, representing 85.6% of all residues. Among these domains, 92% were classified among known folds. To gain insights into the molecular function of fast-evolving MGEs, an expanded comparative analysis of 86 complete *Vpar* genomes was used to define the RIMD mobilome. We highlight domain assignments for pathogenicity island proteins with previously unknown functions and identify previously unrecognized MGEs and fast-evolving proteins that provide a selective advantage for the strain. Domain assignments for the RIMD proteome are provided as a website at http://prodata.swmed.edu/ecod/index_rimd.php.

## RESULTS AND DISCUSSION

### An evolutionary classification of RIMD AlphaFold models improves functional annotation

We classified AlphaFold models from the RIMD proteome into the ECOD hierarchy using an improved version (see Materials and Methods) of the DPAM pipeline (33, 43, 45). DPAM parsed 7,696 domains from 4,822 RIMD proteins, accounting for 85.6% of all residues. Among these, 7,107 (92%) are placed into the existing ECOD hierarchy, accounting for 80.9% of all residues (Fig. S1). The unassigned set includes 589 domains with low DPAM probability (< 0.85), which could either be classified into the existing ECOD hierarchy with manual inspection (half have DPAM probability >0.7) or might adopt new folds not yet cataloged by ECOD. A public database (http://prodata.swmed.edu/ecod/index_rimd.php) provides browsable RIMD domains assigned to the ECOD hierarchy and downloadable data sets of domain ranges, sequences, and structures.

**Fig 1:**
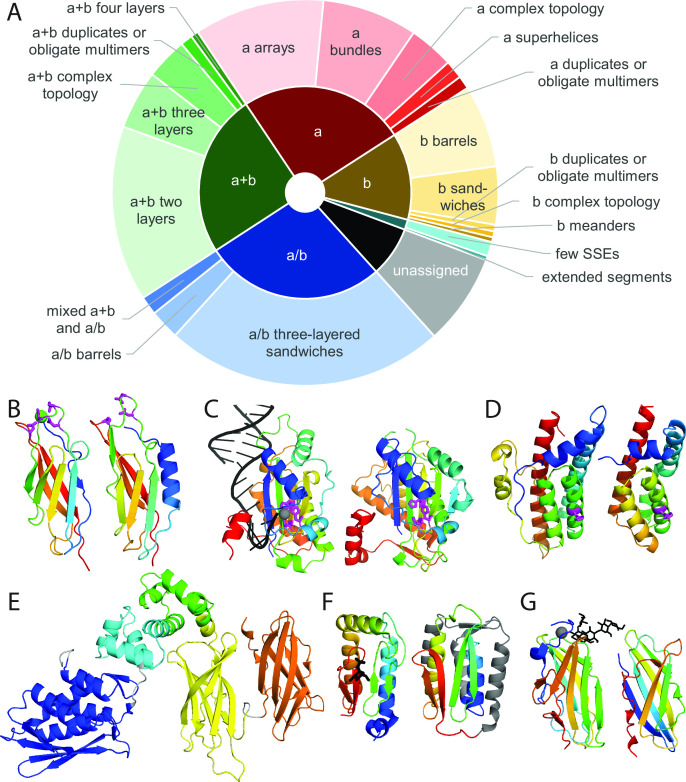
Automated structure classification of RIMD proteome into evolutionary domains. (**A**) A doughnut plot summarizes general fold class and ECOD architectures, which are defined by Secondary Structure Element (SSE) arrangement, parsed and assigned by the DPAM automated pipeline from RIMD AlphaFold models. (**B–D**) Classified RIMD domains (right) and functional insights gained by comparing to their parent domains with confident sequence similarity (left), with all fold topologies depicted in rainbow from the N-terminus (blue) to the C-terminus (red). (**B**) Parent domain (e6dlhA4) coordinates calcium (green sphere) with four residues (magenta stick), three of which are in a domain of VP0053 (magenta stick). (**C**) The parent domain (e4lvlA1) that binds DNA (black cartoon) near active site residues (magenta stick) coordinating Mn (gray sphere) is assigned to a domain of VP2137, which has identical active site residues (magenta stick). (**D**) The parent domain (e2r25A1) includes an active site, histidine, (magenta stick) that is also present in the assigned domain from VPA1046 (magenta stick). (**E**) DPAM parses the VPA1592 AlphaFold model into five domains (blue, cyan, green, yellow, and orange). (**F**) A domain of VPA1592 (right, conserved topology in a rainbow) is assigned to a parent domain in the same H-group as a structure (left, 2fue) bound to mannose 1-phosphate (black stick). (**G**) A domain in VPA1592 (right, rainbow topology) is assigned to the parent domain (1uh4) bound to malto-oligosaccharides (black stick).

A summary of the assigned ECOD domain architectures provides an overview of the fold types in the proteome (Fig. 1A). The most populated architecture, a/b three-layered sandwiches, represents 25% of RIMD domains, significantly higher than the fraction (18%) among non-redundant ECOD domains from experimental structures in the PDB (Table S1). This well-populated architecture includes proteins with a Rossmann-like motif that typically function in metabolism (46). Notably, small protein architectures featured by extended segments and few secondary structure elements are not well populated, as they tend to adopt flexible structures that are harder to classify and are filtered out in the process of identifying globular domains. Filtered sequences are mainly small and flexible, with few secondary structure elements, and often include signal peptides.

Compared to the ECOD PDB domain set (Table S1), the RIMD genome encodes more α-helical domains (27%) than expected (20%). This over-representation reflects an elevated number of transmembrane transporters in the bacterial genome, including dicarboxylate/sodium symporters and ABC transporters. This over-representation could reflect the low fraction of experimentally determined transmembrane proteins (47) but can also signify the ability of bacteria to quickly adapt to their environments by importing or exporting various molecules like sugars, metals, bacteriocins, or antibiotics (48). Multiple bacterial sensing and chemotaxis domains are also over-represented, including HAMP domains and homodimeric domains of signal transducing histidine kinases.

The functions of classified proteins are more meaningful for those with close sequence relationships to known proteins. Among the assigned RIMD domains, 6,476 (90%) were classified with confident sequence relationships to known structures (HHpred probability >90%). While the functions of these proteins are mostly known or can be gleaned from close sequence relationships, a subset (278 proteins, 5.8% of the RIMD proteome) are not annotated with sequence-based domains from the comprehensive database of protein families, PFAM (49), which traditionally provides functional inference for hypothetical proteins. This previously unannotated set of RIMD proteins exemplifies the ability of AlphaFold models to inform function beyond traditional sequence-based methods, as illustrated by the following examples of uncharacterized RIMD proteins.

DPAM parses the VP0053 model into five domains. These include an immunoglobulin-related (Ig) domain with confident sequence similarity (HHpred probability 94%) to a glycoside hydrolase Ig-like calcium-binding domain. The glycoside hydrolase Ig coordinates calcium using four residues. Three are preserved in VP0053, suggesting it might also bind calcium (Fig. 1B). The remaining VP0053 domains (Fig. S2) have diverged beyond sequence recognition and include a duplicated Ig-like domain with similar calcium-binding residues, as well as two interacting lipocalin-like barrels that might bind hydrophobic ligands in the center, similar to the rest of the lipocalin superfamily (50). The last domain possesses an Ig-like topology but lacks a confident automated assignment due to an extension of several helices. VP0053 highlights the ability of AlphaFold models to resolve problems associated with sequence classification of multidomain proteins, especially those with nested insertions.

AlphaFold models also resolve domain assignments for proteins within MGEs that tend to evolve quickly. VP2137 belongs to a *Vpar* pathogenicity island (VPaI), VPaI-4. The model contains a domain classified into the “origin of replication-binding domain fold” in ECOD. The related relaxase MobM binds, nicks, and covalently attaches to the 5′ end of plasmid DNA during conjugative transfer. Three MobM histidine residues coordinate a manganese ion near the active site (Fig. 1C). Their retention in VP2137 suggests a similar function. VPA1046 from the T6SS2 pathogenicity island contains a histidine-containing phosphotransfer domain like the phosphorelay protein YPD1. Preservation of the active histidine in VPA1046 (Fig. 1D) suggests it transfers phosphate to a response regulator, likely to its genome neighbor VPA1045. While AlphaFold models assisted these functional inferences, they could likely be determined by careful manual sequence analysis.

The main novelty of AlphaFold domain assignments is in recognizing distant evolutionary relationships that extend beyond sequence recognition but still inform function. DPAM classified an additional 631 domains into the ECOD hierarchy without significant sequence relationships. These belong to 510 RIMD proteins (11% of the proteome). For example, DPAM parses the VPA1592 AraC-type transcription factor into five domains (Fig. 1E), including a sequence-related AraC-like helix-turn-helix (HTH) duplication. DNA-binding by AraC transcription factors is governed by accompanying companion domains that sense distinct signaling molecules (51). Additional domains in VPA1592 may also function as sensors. One resembles the phosphomannomutase cap domain that recognizes the enzyme’s substrate mannose-1-phosphate (Fig. 1F), while the other two adopt Ig-like folds whose related structures function as carbohydrate-binding modules (Fig. 1G). These relationships suggest a regulatory role for the cap and Ig domains of VPA1592 in sensing carbohydrates.

### Comparison of *Vibrio parahaemolyticus* strains defines the RIMD mobilome

**Fig 2:**
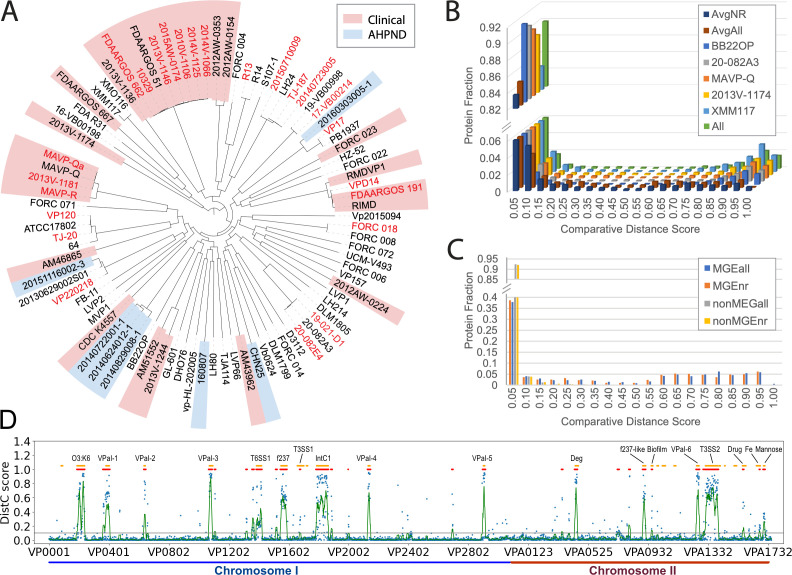
Multiple *Vibrio parahaemolyticus* strain comparisons extend the RIMD mobilome. (**A**) Phylogenetic tree inferred from orthologous sequences representing the core proteome of *vpar* strains with complete genomes. Strains excluded from the non-redundant set are labeled by red fonts. Clades are highlighted according to National Center for Biotechnology Information biosample metadata, with clinical strains in pink and AHPND strains in light blue. (**B**) Histograms depict protein fraction (*Y*-axis) with indicated DistX scores (*X*-axis) for proteins from select proteomes, from all combined strains, and from the average (DistC) across all strains and non-redundant strains. (**C**) Histograms of DistC scores for known MGE proteins and non-MGE proteins, with DistC scores calculated for all proteomes and the non-redundant set. (**D**) DistC scores for RIMD proteins are shown in the same order as encoded in the genome. The blue dots represent the DistC score; the green curve is the smoothed DistC scores by taking the average in windows of 11 proteins (five on each side); the red dots mark smoothed DistC scores above 0.1; the orange dots mark known MGEs. Known MGEs showing high DistC scores (average DistC >0.1) are labeled above the corresponding orange dots, and chromosomes are labeled at the bottom.

The automated assignments described above provide new insights into function, especially for those proteins encoded by known RIMD pathogenicity islands (7–14). These islands are acquired by HGT, allow RIMD to expand ecological and pathogenic niches, and often exhibit fast evolution beyond sequence recognition (20, 41). Previous studies identifying RIMD MGEs (7–14) concentrated on genomic islands and other relatively long sequence regions that typify HGT. However, some RIMD T6SS virulence factors are encoded outside previously discovered MGEs and can exist as smaller auxiliary modules (10, 52). To comprehensively characterize MGEs in RIMD, we compared its proteome to 86 publicly available *Vpar* strains with complete genomes (Table S2). The core genome phylogeny of *Vpar* strains highlights the polyphyletic nature of clinical isolates and acute hepatopancreatic necrosis disease (AHPND) associated shrimp isolates. The disease-causing strains are distributed among non-pathogenic strains in the tree, suggesting that non-core MGEs resulting from HGT lead to pathogenicity (Fig. 2A).

To help identify RIMD virulence factors, we compared the proteome to those encoded by 86 *Vpar* strains. A comparative distance score, DistX (see Materials and Methods), was computed for each RIMD protein, reflecting its divergence from the closest protein sequence in each of the other strains. DistX is 0 if another *Vpar* proteome possesses an identical sequence as a RIMD protein; DistX is 1 if this protein is absent or not detectable in another *Vpar* strain due to fast evolution. DistX scores for RIMD proteins compared against other *Vpar* strains follow a bimodal distribution with two peaks around 0 and 1, respectively (Fig. 2B). DistX scores are mostly below 0.05, reflecting the similarity between RIMD and other strains. We used the fraction of RIMD proteins with DistX of <0.05 when compared against another *Vpar* strain to measure its distance from RIMD. Using this estimate, results showed that the pre-pandemic BB22OP strain (53) is the closest environmental isolate to RIMD (91.5%) among our data sets, despite the two being more distant in the phylogenetic tree of the core genome (Fig. 2A). This similarity is due to an overlap of accessory proteins in the two strains and primarily reflects the presence of the same secretion systems: two T3SS and two T6SS are shared by the RIMD and BB22OP strains. Two selected clinical strains, MAVP-Q (90.1%) and 2013 V-1174 (89.0%), and a strain isolated from AHPND shrimp, 20–082A3 (90.9%), also have similar DistX distributions as BB22OP. A strain isolated from seawater, XMM117, lacks T3SS2, with T6SS1 diverging more than the strains mentioned above. Its DistX distribution shifts toward higher values and lower fraction below 0.05 (84.8%).

DistX scores for each of the 86 strains reveal similar bimodal distributions, suggesting a presence (DistX close to 0) or absence (DistX close to 1) scenario for most of the RIMD proteome. Elevated DistX indicates HGT or fast evolution, and a composite distance score summarizing all strains should indicate the degree of these evolutionary events for each RIMD protein. We calculated the average of DistX, namely, DistC, across all 86 strains and a subset of 64 strains after removing redundancy, respectively. DistC also adopts a bimodal distribution, but the scores are more dispersed (dark blue and maroon bars in Fig. 2B). This dispersed distribution suggests that *Vpar* strains experience different evolutionary events, and the gain and loss of genes vary between strains. We considered RIMD proteins with DistC below 0.05 present in almost all strains. Roughly 15% (721 out of 4822) of the RIMD proteome show DistC above 0.05 and mostly represent either MGEs or fast-evolving genes with respect to the other strains.

To test whether DistC scores can be used to identify the RIMD mobilome, we examined the distribution of DistC for known RIMD MGEs. The DistC scores for MGE-encoded proteins shift to higher values than the non-MGE DistC scores (Fig. 2C), with ~60% showing DistC above 0.05. Nineteen out of the 26 known MGEs show an average DistC above 0.05, and MGEs with an identified integrase or transposon show much higher DistC (above 0.4, as in Table 1). Despite the lack of an integrase/transposon, the O3:K6 antigen region encoding the capsular polysaccharide (CPS) proteins and transport apparatus also shows a high average DistC (0.46). This region is reported to evolve by recombination and gene duplication (54, 55). Regions showing high DistC (average DistC above 0.1 in a window of 11 consecutive proteins) mostly overlap with known MGEs (Fig. 2D). However, we found additional, mostly shorter regions in the RIMD genome showing elevated DistC scores (discussed in Novel Mobile Genetic Elements Identified in the RIMD Mobilome).

**TABLE 1:** Previously identified *Vibrio parahaemolyticus* RIMD MGEs

RIMD Open Reading Frames (ORF)s	Region type	Int^*a*^	DistC_avg_	PubMed reference number (PMID)
Chromosome I				
VP0081–VP0092	NK	–	0.02	18590559
VP0187–VP0238	O3:K6 Antigen	–	0.46	18195030
VP0380–VP0403	VPaI-1	Int	0.516	16672049
VP0634–VP0643	VPaI-2	Int	0.447	16672049
VP1071–VP1095	VPaI-3	Int	0.582	16672049
VP1386–VP1420	T6SS1	–	0.341	18590559
VP1549–VP1590	Phage f237	Int	0.49	9399511
VP1658–VP1702	T3SS1	–	0.04	16672049
VP1719–VP1728	Osmotolerance	–	0.017	16894340
VP1787–VP1865	Integron class-1	Int	0.519	18590559
VP2131–VP2144	VPaI-4	Int	0.672	16672049
VP2900–VP2910	VPaI-5	Int	0.813	16672049
Chromosome II				
VPA0434–VPA0458	Degradative	Int	0.438	18590559
VPA0887–VPA0914	Phage f237-like	Int	0.418	18590559
VPA0950–VPA0962	Biofilm	–	0.129	18590559
VPA0989–VPA0999	Gametolysin	–	0.085	18590559
VPA1024–VPA1046	T6SS2	–	0.015	22924031
VPA1102–VPA1115	Osmotolerance	–	0.023	16894340
VPA1253–VPA1270	VPaI-6	Int	0.662	16672049
VPA1312–VPA1395	VPaI-7 (T3SS2)	Tnp	0.583	16672049
VPA1403–VPA1412	CPS	–	0.026	18590559
VPA1440–VPA1444	T1SS	–	0.074	18590559
VPA1503–VPA1521	Type I pilus	–	0.02	18590559
VPA1559–VPA1583	Multidrug efflux	–	0.091	18590559
VPA1652–VPA1679	Ferric uptake	–	0.07	18590559
VPA1700–VPA1709	Mannose metabolism	–	0.309	18195030

^*a*^ Presence of an integrase (Int), transposase (Tnp), or none (–) according to a previous study (7).

### RIMD MGEs are enriched with phage proteins and bacterial defense domains

**Fig 3:**
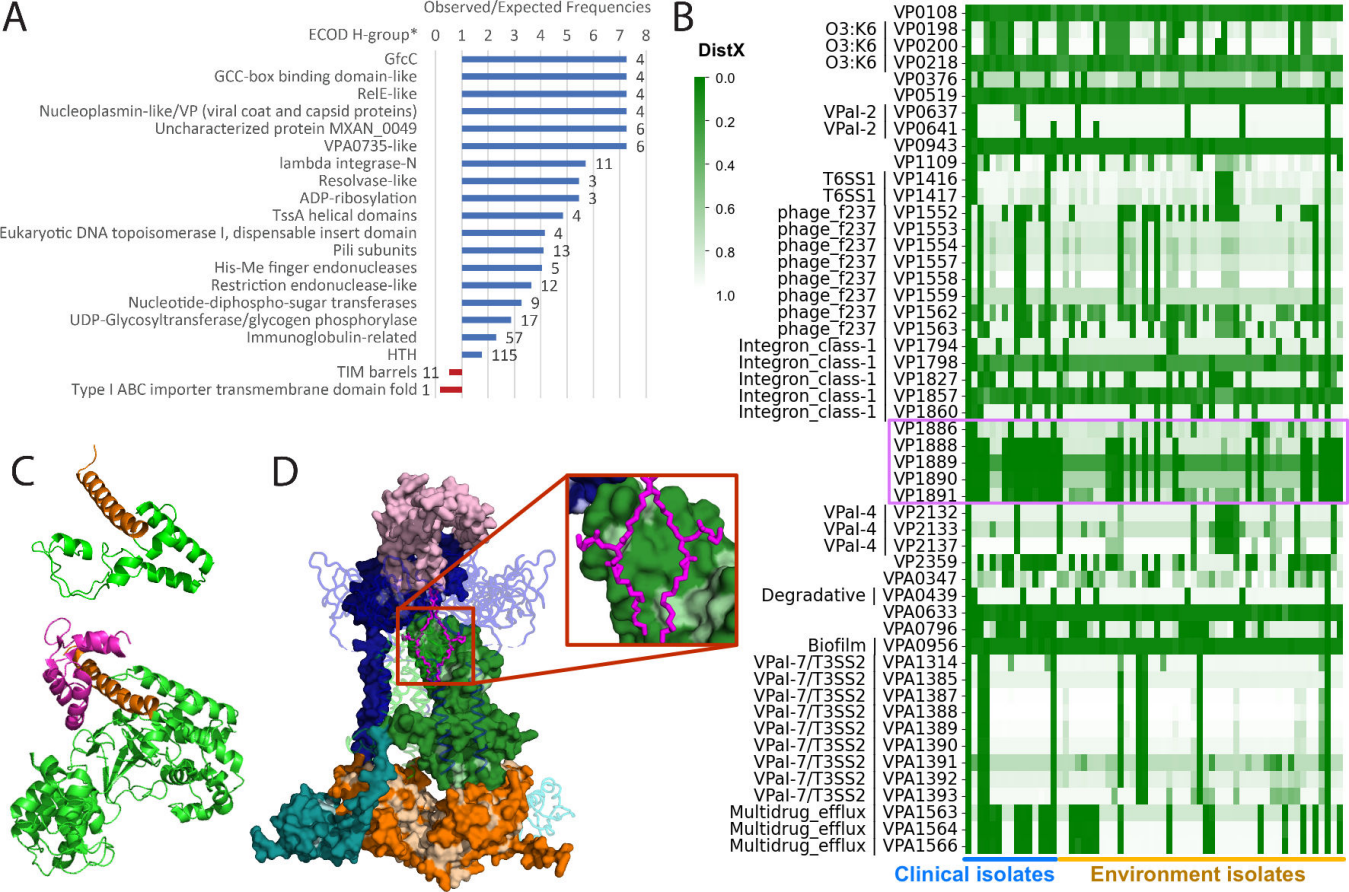
Domain assignments for the RIMD mobilome. (**A**) Bar graph of observed/expected frequencies calculated for H-groups (labeled on the left) of RIMD domains from its mobilome (compared to all assigned domains). This graph includes over-represented and under-represented H-groups with significance of <0.05 by Fisher’s exact tests and MGE domain count of >2. (**B**) Heatmap of DistX scores among non-redundant proteomes for proteins whose frequency of being present and conserved (DistX <0.05) in clinical isolates is at least two times the frequency in environmental isolates. We colored high DistX by lighter green because a high score frequently indicates the protein is absent from a proteome. (**C**) The ospC2-like sequence in VPA1331 (top) is a deteriorated fragment of OspC2 (bottom, green cartoon, 7wzs) which binds host calmodulin (magenta) using an N-terminal helix (orange) that is retained in VPA1331. (**D**) Surface rendered AlphaFold model of MlaDEF-like putative ABC transport system (VP1361-VP1364), where color code distinguishes different domains. Amino acid positions constant between this system and a classic MlaBCDEF system of RIMD (VP2660-VP2664) are colored light, and variable positions are colored dark. These models are superimposed onto an ABC transporter (PDB: 7cge) multimeric assembly shown as a tube and colored by chain. A phospholipid bound to the ABC transporter is shown as magenta sticks and zoomed in in the insert.

We consider the set of proteins with DistC above 0.05 as the RIMD mobilome that potentially originates from HGT, and we thus refer to them as HGT proteins/domains. Figure 3A highlights which types of domains are over-represented in the RIMD mobilome compared to the rest of the proteome. The enriched HGT domains include homologous groups that typically belong to phages or mediate DNA exchange, such as nucleoplasmin-like/viral coat and plasmid proteins, lambda integrase domains, resolvase-like, His-Me finger endonucleases, and phage-related HTH DNA-binding domains (Fig. 3A). The presence of phage-related proteins highlights their involvement in passing DNA from one bacterial host to another by various documented mechanisms (56). Prophage-related elements can thus indicate passenger genes acquired in bacterial genomes and contribute to antibiotic resistance and pathogenicity (56).

Additional over-represented domains among the RIMD mobilome include toxin-antitoxin (TA) systems that play roles in bacterial adaptation to phage infection, antibiotics, or oxidative stress. RNA cleavage by RelE-like toxins is inhibited by interaction with extended C-termini of the more diverse antitoxins. The antitoxin N-termini adopt different DNA-binding folds, such as HTH in RelB-like and an α + β domain in the YefM-like antitoxins. These N-domains regulate their respective TA operons. Three RelE-like TA systems reside in the RIMD integron-class1 (IntC1) pathogenicity island (VP1787–VP1865): VP1820/VP1821 (YefM-like), VP1830/VP1829 (RelB-like), and VP1843/VP1842 (RelB-like), highlighting the ability of this region to acquire mobile gene cassettes through its recombination system.

Another category of over-represented HGT domains includes restriction-modification systems (restriction endonuclease-like, His-Me finger endonucleases, and HTH domains) involved in defense against phage (57). RIMD includes 12 HGT proteins with a restriction endonuclease-like fold, with 10 encoded by the known pathogenicity islands: VPaI-1 (VP0395, VP0400, and VP0401), VPaI-3 (VP1083 and VP1087), int-C1 (VP1805 and VP1823), VPaI-4 (VP2143), VPaI-6 (VPA1256), and multidrug efflux (VPA1572). Two restriction enzymes include P-loop NTPase domain helicases like the newly described nuclease-helicase immunity (Nhi) family that targets and degrades phage-specific replication intermediates (58). One putative Nhi (VP1083) includes a zincin-like metalloprotease domain and may also cleave proximal DNA-binding proteins, while the other (VP0395) includes HTH domains that likely bind DNA. In other bacterial pathogens, restriction-modification systems have been implicated in virulence and host immune evasion through methylation-controlled phase variation, i.e., the switch of gene expression profiles (57). The VPaI-1 region includes two proteins (VP0388 and VP0394) with S-adenosylmethionine-dependent methyltransferase domains that might play similar phase variation roles.

Finally, several domains involved in modifying sugars (GfcC, UDP-glycosyltransferase/glycogen phosphorylase, and nucleotide-diphosphate-sugar transferases) of bacterial lipopolysaccharide (LPS, O antigen) and CPS (K antigen) are over-represented among the RIMD mobilome. These groups stem from the known MGE encoding O3:K6 antigen determinants (14), which are responsible for stimulating innate immunity in humans and are targets of antibacterial drugs. The region is known to include a small set of conserved core genes that likely synthesize the common LPS precursor and a large set of variable genes among 40 *Vpar* K serotypes (55). Gene duplication, such as an expansion of glycosyltransferases, drives the evolution and diversification of the region (55).

### Patterns of presence/absence for RIMD proteins in known MGEs suggest virulence

Proteins encoded by known MGEs may contribute to the pathogenicity of RIMD. We identified 51 proteins showing higher preference to be present and conserved among non-redundant clinical strains than environmental isolates (and see Materials and Methods), and 37 of them are encoded by known MGEs (Fig. 3B). These proteins include a thermostable direct hemolysin (VPA1314) and the CRISPR/Cas system (VPA1388-VPA1390) from the VPaI-7 (T3SS2). Consistent with this observation, a previous comparative genomic study proposed that the T3SS2 pathogenicity island in a related MAVP-Q strain was acquired independently to gain pathogenicity from a previously benign strain (59). Additional clinical strain-associated proteins are encoded by the filamentous phage f237, whose fragment encoding VP1561 has been used as a genetic marker for pandemic strains (60), and the IntC1 region (Fig. 3B). The IntC1 phage integrase (VP1865) includes a lambda-integrase N-terminal domain followed by two C-terminal HTH domains. Similar integrases mediate recombination between an integrase proximal primary site (attI) and a secondary target site (attC) found within mobile gene cassettes encoding resistance or virulence factors (61). Thus, integrons like IntC1 play a major role in the spread of multidrug resistance and other virulence mechanisms. Clinical isolate segregating proteins acquired by this region include a potential glyoxalase/bleomycin resistance protein (VP1798) and two acetyltransferases (VP1794 and VP1827) that could potentially modify antibiotic peptides, small molecules, or other proteins.

Our domain classification of RIMD proteins provides functional insights into known MGEs without well-defined functions (Table 1). Despite the challenge of classifying such proteins, we could confidently assign domains in 483 (73%) out of 663 (Table S3). As suggested by the enrichment of RelE-like TA systems and restriction-modification system domains among MGEs (Fig. 3A), the inferred functions of many other MGE-encoded proteins might provide a selective advantage to RIMD. For example, VPaI-1 encodes a high-persistence TA system that regulates the formation of persister cells to survive various stresses, including antibiotics (62), and VPaI-4 encodes an AAA +ATPase-containing protein (VP2142) with an adjacent MrcB-like restriction enzyme (VP2143). Homologous AAA + ATPase and restriction enzyme systems cleave invading methylated phage DNA (63). Acquisition of these two pathogenicity islands by RIMD likely confers phage resistance to the strain, providing a potential selective advantage over competing bacteria as well as allowing persistence in stressful environmental niches like those encountered in the host gut.

Similar patterns of presence and absence across *Vpar* strains suggest the VPaI-2 and Deg islands might function together. While VPaI-2 encodes phage machinery, Deg includes a T3SS1 chaperone (VPA0451) and a T3SS1 effector inositol phosphatase (VPA0450) that functions in virulence (64). The position of VPA0450 in Deg instead of the T3SS1 island suggests the chaperone/effector pair may have been acquired independently of these T3SS1 machinery (the more ancient of the two T3SSs). Alternately, VPaI-5 and VPaI-6 are mainly limited to RIMD and closely related strains. VPaI-6 encodes a protein with a nuclease-like colicin domain (VPA1263) whose homolog can kill sensitive bacterial cells by translocating into their cytoplasm and cleaving DNA (65). This acquired bacterial competition system is analogous to the role of T6SS, which injects toxic proteins into target bacteria to gain a competitive advantage.

The T6SSs were previously analyzed using pan-genome comparisons, which highlighted acquisition of the T6SS1 by HGT and omnipresence of the T6SS2 (10). Consistent with this finding, proteins encoded by T6SS1 have elevated DistC, while proteins encoded by T6SS2 have low DistC. Notably, the annotated T6SS2 island appears to be restricted to the T6SS secretion machinery, auxiliary, and regulatory proteins, with identified effector immunity pairs residing outside of the T6SS2 genomic neighborhood (66). The T6SS2 includes a single hypothetical protein of unknown function (VPA1024). VPA1024 adopts a duplicated thiolase-like fold, with the N-terminal domain having similarity to a non-canonical ketosynthase FabY and the C-terminal domain having similarity to a fatty acid synthase alpha subunit. Many thiolase-like folds represent modules of polyketide synthases, whose metabolites include diverse chemical structures and biological activities including antibiotic and predator defense properties (67). The association of polyketide or fatty acid modification with the T6SS remains enigmatic, but an unknown protein (VP1399) belonging to the more recently acquired T6SS1 adopts a similar thiolase-like fold duplication.

Numerous fragmented proteins in RIMD genome MGEs have high DistX due to their small size. One such protein, VPA1331, encoded by the T3SS2 resembles a portion of the outer *Shigella* protein family protein OspC2. Full-length OspC2 includes a catalytic domain that modifies host protein Arg side chains with ADP-riboxanase (68). The structure of a related *Chromobacterium violaceum* effector CopC highlights its interaction with host calmodulin and caspase-7 (60). While VPA1331 does not possess the entire catalytic domain for activity, it retains the N-terminal helix responsible for binding calmodulin in the Ca^2+^-free state (Fig. 3C). Thus, the OspC2 deterioration in VPA1331 may retain host calmodulin-binding activity, preventing its association with Ca^2+^. The T3SS2 confers RIMD the ability to invade host epithelial cells. Survival within intracellular niche allows the bacteria to evade the extracellular host immune system and antibiotics. Potentially, maintenance of this calmodulin-binding fragment aids in RIMD survival inside host cells.

### Novel mobile genetic elements identified in the RIMD mobilome

Given the tendency of proteins from known MGEs to exhibit elevated DistC values, we propose that genes with similar elevated DistC might also be mobile. Potential novel MGEs with DistC above 0.05 encode an additional 314 proteins. We assigned 444 domains from 235 of these proteins (Table S4). These MGEs form small clusters: *VP0363–VP0377, VP1355–VP1370, VP1517–VP1522, VP1884–VP1891, VP2692–VP2698, VPA0713–VPA0735, and VPA0790–VP0800*.

*VP1361–VP1364* encodes a putative ABC transport system inserted into the RIMD genome with respect to the pre-pandemic strain BB22OP. An experimental structure of the classic MlaBDEF allows assembly of the HGT system by superimposing the component structures (Fig. 3D; VP1361–VP1364 in surface representation, experimental structure in ribbon). The HGT system includes a MlaE-like permease (VP1361, green), MlaF-like ATP-binding cassette (VP1363, orange), and a MlaD-like periplasmic component (VP1364, blue). The orthologous MlaBCDEF system present elsewhere in the RIMD genome (VP2660–VP2664) maintains cell membrane integrity by transporting glycerophospholipid. The HGT system has diverged from the classic RIMD MlaE (VP2663) and MlaF (VP2664), retaining 27% and 37% sequence identity, respectively. The classic MlaB (VP2660) SpoIIaa-like domain is instead fused to the HGT MlaE-like VP1361 (Fig. 3D, teal), and the periplasmic HGT MlaD-like VP1364 has an additional helical domain at the C-terminus (Fig. 3D, pink), replacing the presumed extrusion function of the classic MlaC (VP2661). A zoom of the HGT MlaE-like permease superimposed with the MlaBDEF structure phospholipid (Fig. 3D insert, magenta) reveals partial conservation of the interior glycerophospholipid tunnel, suggesting the system might transport an alternate lipid substrate.

The region *VP1884–VP1891* contains several genes preferably associated with clinical strains (Fig. 3B). *VP1888–VP1891* are present in 87% (14 out of 16) of clinical strains and 30% of environmental strains, suggesting that this region might be related to pathogenicity. This region encodes an OB-fold cold shock protein (VP1889) whose homologs work as RNA chaperones, and an RNase R 3′−5′ exoribonuclease (VP1890) typically involved in the maturation of structured RNAs. The specific roles of these proteins in RIMD’s adaptation and pathogenicity remain to be explored.

A neighborhood from *VP0363* to *VP0377* with elevated distances (8 out of 15 genes show DistC >0.05) encodes a five-gene cassette that includes glycerol dehydrogenase (VP0363), dihydroxyacetone kinase subunits (VP0364 and VP0365), phosphoenolpyruvate-protein phosphotransferase PtsA (VP0366), and a dihydroxyacetone kinase operon transcriptional regulator DhaR (VP0367). This cassette may encode proteins responsible for RIMD utilization of glycerol as a carbon source in anaerobic conditions (69). These examples of potential MGEs support the role of mobile proteins in virulence and other functions that confer a selective advantage for the bacteria.

### Orthologous genome neighborhoods distinguish fast-evolving proteins from HGT

Elevated DistC scores do not distinguish between proteins arising from HGT and those evolving rapidly. To better discriminate between these events, we compared the RIMD genomic neighborhood of each protein to its neighbors in the pre-pandemic environmental BB22OP strain. A protein displaying an elevated DistX (>0.05) score between RIMD and BB22OP but with similar genomic neighbors in both strains was considered as fast-evolving. Among 102 fast-evolving proteins (Table S5), 132 domains in 73 are confidently classified.

The elevated DistX scores may reflect differences in protein lengths (i.e., Fig. 3C), lowered sequence identity, or a combination of both. For example, the RIMD effector VopV (VPA1357) from the T3SS2 pathogenicity island is twice the size (1622 residues) of the orthologous sequence (VPBB_A1234, 876 residues) in the BB22OP T3SS2. Both sequences include intrinsically disordered glycine-rich repeats. Another fast-evolving RIMD protein (VPA1455) includes a 66-residue polyQ repeat that is longer than the corresponding BB22OP protein (39 residues). An orthologous protein from *V. cholera* acts as a receptor for phage, and decreased length of the polyQ dictates natural phage resistance (70). These examples highlight the ability of low complexity repeat sequences to diverge quickly between strains due to fewer structural constraints.

**Fig 4:**
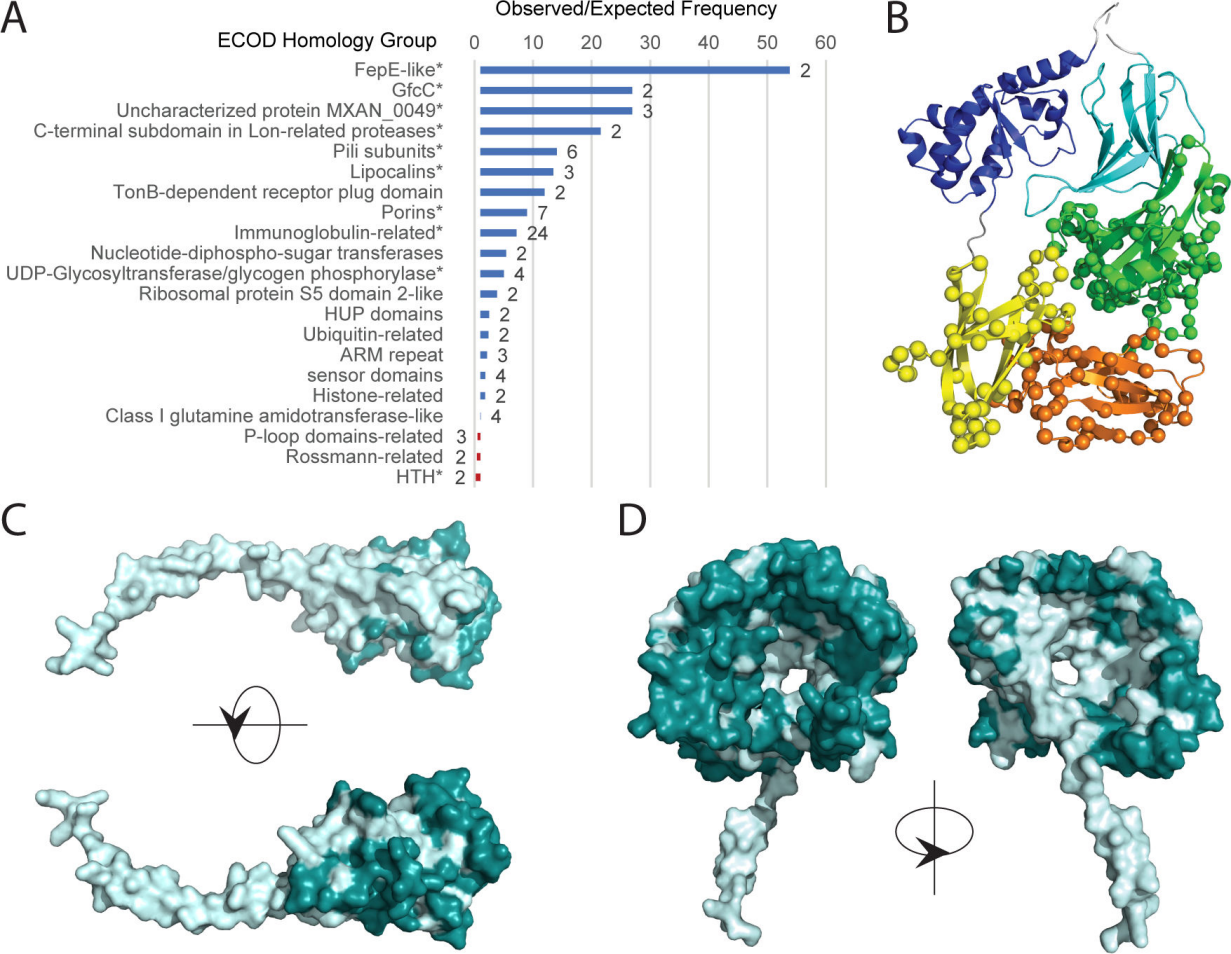
*Vibrio parahaemolyticus* genome neighborhood comparison reveals fast-evolving proteins (**A**) Bar graph of observed/expected frequencies calculated from fast-evolving RIMD domains (expected frequencies are calculated using all assigned domains from the RIMD proteome) assigned to ECOD H-groups labeled on the left. All H-groups with more than one assigned domain are included. Significance defined as a *P* value of <0.05 by Fisher’s exact test is noted as * after the labels. (**B**) A secreted T6SS co-effector, VP1388, colored by domains, with variable amino acids compared to the ortholog from BB22OP marked by spheres. (**C**) MshA-like pilin subunit, VP0747, displayed in surface representation, with constant amino acid positions between RIMD and BB22OP colored light cyan and variable positions colored dark teal. The upper panel is rotated 180° along the *X*-axis with respect to the lower panel. (**D**) A maltoporin, VPA1644, colored as in panel **C** but rotated 180° along the *Y*-axis.

To determine over-represented domain types among these fast-evolving proteins relative to the entire RIMD proteome, we performed enrichment analysis for ECOD homologous groups appearing more than once in the fast-evolving protein set (Fig. 4A). This comparison revealed pili subunits, lipocalins, immunoglobulin-related, and porins, among others. Typically, such domains mediate interactions at the bacterial cell surface that might evolve to adapt to environmental changes and evade unfavorable factors such as the host immune system. Therefore, analysis of fast-evolving RIMD proteins can provide insights into RIMD’s adaptation to their environment.

### Fast-evolving RIMD proteins promote competitive traits

The T6SS1 pathogenicity island in RIMD and BB22OP strains injects toxic proteins into target bacteria, fungi, or host cells to gain a competitive advantage. The T6SS1 includes an operon consisting of a secreted co-effector (VP1388), an immunity protein (VP1389), and a coregulated toxin (VP1390) responsible for antibacterial toxicity (71, 72). All three proteins diverged rapidly between RIMD and BB22OP (DistX >0.4). Divergence in VP1388 is not uniform across the protein structure (Fig. 4B, spheres depict differences). The conserved N-terminus includes a deteriorated helix-extension-helix motif followed by an immunoglobulin-related domain representing part of a MIX secretion signal (72). Three divergent C-terminal immunoglobulin repeats resemble structures that mediate bacterial adhesion (VP1388 domain 3 and 4) and carbohydrate recognition domains (VP1388 domain 5). The fast-evolving C-terminal domains are likely co-evolving with the interacting toxin, while the invariant MIX secretion signal interacts with the conserved T6SS1 machinery.

Several RIMD pili domains are experiencing fast evolution, including chitin-regulated pilus PilA (VP2523), mannose-sensitive hemagglutinin (MSHA) biogenesis pilus (VP2696), and an isolated pilin (VPA0747). The globular heads of pili have been shown to undergo rapid adaptation to accommodate variation at the recipient cell surface for functioning in antigenicity, adhesion, and colony formation (73). Accordingly, the MshA pilus of RIMD binds human host intestinal endothelial cells, while both PilA and MshA mediate biofilm formation and adherence to chitin polymers abundant in estuarine waters serving as *Vpar’s* environmental niche (74, 75). The isolated MshA-like pilin VPA0747 in RIMD and BB22OP share 67% identity, with variations concentrated in the globular head (Fig. 4C**,** identical light cyan and variable dark cyan). Another cluster of four uncharacterized proteins with pili domains include two fast-evolving proteins: VP0658 (DistX: 0.71) and VP0659 (DistX: 0.84) that might also be involved in recognition of other cells or abiotic surfaces.

Multiple outer membrane porins are also experiencing fast evolution, including maltoporin (VPA1644) and its two neighboring genes, maltose periplasmic protein MalM (VPA1642) with an assigned concanavalin A-like domain, and a presumed periplasmic protein with a starch specific carbohydrate-binding module (VPA1643). Interestingly, maltoporin also functions as a lambda phage receptor. Thus, the rapid evolution might be due to selection against this co-habiting phage. In support of this hypothesis, residues altered in the maltoporin line the extracellular surface of the protein, while conserved residues line the pore and the periplasmic surface (Fig. 4D, identical light cyan and variable dark cyan). Thus, the fast-evolving structure may maintain the essential maltose-transporting function of the protein while masking its surface from phage.

Several other fast-evolving domains belong to the RIMD O3:K6 antigen or CPS islands that determine the composition of the polysaccharides on the bacterial surface. These domains include homologs of the bacterial polysaccharide co-polymerase FepE (VP0221 and VPA1406) (76), the GfcC protein (duplicated in VP0216) that is essential for assembly of the O-antigen capsule and may be necessary for secreting biofilm-forming exopolysaccharides (77), and the UDP-glycosyltransferase/glycogen phosphorylase (VP0211 and VP0212) enzymes that transfer activated sugars to a variety of substrates. The fast evolution of these LPS island proteins likely correlates with altered substrate specificity for polysaccharides in RIMD (O3:K6) compared to BB22OP (O4:K8).

### Conclusions

Evolution-based domain classification established in ECOD provided the basis for enhanced functional insight into the RIMD proteome. Results are deposited as an online database for further investigation by the scientific community. Comparisons with complete *Vpar* strains revealed the RIMD mobilome, which overlaps with known MGEs but also includes novel HGT proteins. Evolution and structure-guided functional inference for the RIMD mobilome revealed enrichment of phage domains that mediate HGT and domains used in bacterial defense. Classified domains revealed potential roles in RIMD’s responses to human hosts, bacterial phages, and other stresses. Finally, comparison with a pre-pandemic strain identified fast-evolving proteins that may contribute to RIMD’s virulence and adaptability. These results provide valuable insights into bacterial evolution, pathogenic mechanisms, and adaptation strategies of *Vpar* while offering testable hypotheses for future experimental studies. Overall, our research contributes to a deeper understanding of the complex mechanisms of *Vpar* pathogenicity and sheds light on potential targets for therapeutic interventions.

## MATERIALS AND METHODS

### Domain parsing and classification of the RIMD proteome

AlphaFold models from the proteome of *Vpar* RIMD (TaxID: 223926) were downloaded from the AFDB Google Cloud repository in August 2022 (v.3). Of these 4,800 models, 325 were affected by errors in modeling that were fixed in a later release; we thus replaced this subset with the updated v.4 models (42). Similar to those described previously (44), the 4,800 models were partitioned into domains and classified (where possible) against the ECOD domain classification using the DPAM (43). DPAM is our recently developed classification pipeline that relies on ECOD hits found by HHsuite and Dali (78, 79), as well as inter-residue distances and predicted aligned errors of AlphaFold models to partition structures into compact domains. Each parsed domain was assigned to the ECOD hierarchy by finding an ECOD parent domain showing the highest DPAM probability for being in the same ECOD T-group as the query domain. The DPAM probability was calculated based on sequence and structure similarities evaluated by HHsuite and Dali and the consensus between them, as detailed in our previous work (44).

In this study, we discovered that DPAM might split the decorations of core domains as separate domains because these decorations are frequently not tightly packed against the cores. Therefore, we modified our DPAM pipeline to examine neighboring domains assigned to the same ECOD T-group and decide if they should be merged into a single domain. Neighboring domains are detected by sequence (separated by ≤5 residues) and three-dimensional structure (with ≥9 residue pairs that are ≤8 away). We counted the fraction of ECOD hits with confidence comparable to top hit (DPAM probability > top DPAM probability + 0.1) that support merging two neighboring domains, i.e., both query domains mapping to different regions of the same ECOD domain with overlap of <25% of the mapped residues for either query domain. We merge the two neighboring domains if this fraction is above 50%.

Additionally, domains detected by the DPAM pipeline were further evaluated by the number of secondary structure elements [≥6 residues predicted as H, G, or I by DSSP (80) were considered a helix, and ≥3 residues predicted as E or B by DSSP were considered as a strand] and the completeness of a domain. A domain with less than three secondary structure elements and without confident (HHsuite probability ≥95% and coverage ≥80%) ECOD hits by HHsuite is considered a “simple topology.” A domain that is too short compared to its ECOD parent domain (<1/3 residues aligned) or the median length of the ECOD T-group (<1/3 length) it is assigned to is considered a “partial domain.” Domains passing these filters are regarded as “low confidence” and cannot be automatically assigned if their DPAM probabilities are below 0.85, while the rest are considered “good domains” with confident assignments. The statistics of domains belonging to each category are shown in Fig. S1. The set of good domains are presented and studied subsequently. We presented the results as an online database using the ECOD framework that operates on a PostgreSQL database which serves a PHP-based F3 front end.

We compared the distribution of good domains in the RIMD proteome among each ECOD A-group against experimental structures filtered by 99% sequence identity (PDB F99 set). The fold enrichment of RIMD domains in an A-group is calculated as the ratio between the fraction of the RIMD domain in this A-group and the fraction of domains from the PDB F99 set in the same group. The statistical significance for enrichment or depletion (Table S1) of RIMD domains in an A-group is evaluated by Fisher’s exact tests (scipy.stats.fisher_exact).

### Identifying the *Vibrio parahaemolyticus* RIMD mobilome and fast-evolving proteins

GenBank (GCA) and RefSeq (GCF) genomes for *Vpar* strains were filtered for complete assemblies annotated by RefSeq (86 strains released before 22 February 2023 as in Table S2), and their protein sequences were downloaded from National Center for Biotechnology Information (NCBI) data sets. Phylogeny of these *Vpar* strains was inferred from aligned orthologous sequences using the M1CR0B1AL1Z3R server (81). Protein sequences corresponding to the AFDB models were downloaded from Uniprot (UP000002493) with locus gene names. The Uniprot RIMD proteome was compared to the GenBank and RefSeq RIMD annotations using BLAST to identify identical sequences of the same length.

We searched every Uniprot RIMD protein against each *Vpar* strain by BLAST (E-value 1,000) and extracted the bitscore from the top hit. A comparative distance score (DistX) for each protein against each proteome was calculated from the top hit bitscores from the RIMD (Refbs) proteome and the top hit bitscore of this proteome (S1bs) as dX = |S1bs – Refbs| / Refbs. Such comparisons could be affected by differences in gene annotation pipelines. Thus, we selected the *Vpar* strain RefSeq proteomes consistently defined by the NCBI prokaryotic genome annotation pipeline (82). However, comparison of the RIMD RefSeq and author annotated GenBank proteomes (Fig. S3A) to AlphaFold model sequences suggests the GenBank proteome shares more identical sequences than the RefSeq proteome, but the latter is more appropriate for strain comparisons. Inspection of inconsistently defined RIMD proteins between RefSeq and GenBank revealed that most inconsistencies (605 proteins) arose from the alternately defined start or stop sites, and the rest (395 proteins) were excluded or annotated as pseudogenes in RefSeq due to their small size. To minimize the influence of discrepancies between the Genbank and RefSeq annotations of RIMD, we calculated DistX using both sets of annotations, keeping the smaller of the two (Fig. S3B).

We averaged the DistX scores for different *Vpar* strains to obtain DistC, an indicator of fast-evolving or horizontally transferred genes absent in many strains. Because strain redundancy may influence the DistC scores, we eliminated those strains whose DistX scores for the whole proteome had Pearson’s correlation coefficients (using the CORREL function in Excel) above 0.95, resulting in 64 non-redundant *Vpar* strains. We calculated DistCnr using this non-redundant set. However, DistC and DistCnr are highly correlated with a Pearson’s correlation coefficient of 0.998, suggesting that our analysis is robust to the redundancy in the *Vpar* proteome. Thus, we relied on DistC in this work, and RIMD proteins with DistC above 0.05 (determined empirically from DistC distribution) are considered among its mobilome.

To distinguish between fast evolution and HGT for RIMD proteins showing elevated distances between strains, the genome neighborhood of each RIMD protein was compared to that of the BB22OP strain. We compared these two strains using The Rapid Annotation of microbial genomes using Subsystems Technology (RAST) server (RASTtk annotation scheme, fix errors, fix frameshifts, and backfill gaps) with the SEED database sequence-based comparison (83, 84). Fast evolution was defined as the RIMD protein having DistX of >0.05 against the BB22OP proteome and equivalence between the RIMD and BB22OP genome according to the RAST genome neighborhood viewer (83, 84). Some RIMD proteins with elevated BB22OP-specific DistX were filtered out due to short and/or low complexity structure.

### In-depth analyses of the RIMD mobilome and fast-evolving proteins

We compared RIMD mobilome against the known MGEs (7) by plotting the DistC scores for proteins in the order encoded by the RIMD genome using Jupyter Notebook (Fig. 2D). Clusters of proteins with elevated DistC scores were identified by computing the average DistC scores in windows with 11 proteins (adding five on each side of a protein). To identify RIMD proteins that tend to be present and conserved (DistX <0.05) among clinical isolates, we classified the non-redundant *Vpar* strains based on NCBI Biosample metadata into clinical and environmental (environmental, aquatic, and sediment) isolates. The fraction of clinical isolates with a protein (DistX <0.5) is compared against the fraction of environmental isolates, and those with the first ratio twice the second are shown in Fig. 3B.

Proteins of the RIMD mobilome were studied manually using the AlphaFold models and with the help of ECOD assignments and the ECOD parent domains derived from experimentally characterized PDB entries. We analyzed the distribution of RIMD domains of proteins in the RIMD mobilome among different ECOD H-groups using all domains in the RIMD proteome as the background. Fold enrichment in an H-group is calculated as observed/expected frequencies: ${(Cmob}_{Hgrp}/{Cmob}_{all})/{(Cpro}_{Hgrp}/{Cpro}_{all})$. ${Cmob}_{Hgrp}$ and ${Cmob}_{all}$ are the counts of domains from the RIMD mobilome in this H-group and in all H-groups (i.e., total mobile domains), respectively; ${Cpro}_{Hgrp}$ and ${Cpro}_{all}$ are the counts of domains from the entire RIMD proteome in this H-group and in all H-groups (i.e., total domains in the proteome), respectively. Statistical enrichment is computed by comparing ${(Cmob}_{Hgrp}:{Cmob}_{all})$ against ${(Cpro}_{Hgrp}:{Cpro}_{all})$ using Fisher’s exact function in Excel (hypgeom.dist) and a *P* value cutoff of 0.05. Similarly, we analyzed fast-evolving proteins and computed enrichment of H-groups among these proteins, and we ignored H-groups represented by a single fast-evolving domain.

## SUPPLEMENTAL MATERIAL

Tables S1 to S5 msystems.00796-23-s0001.xlsx Tables for RIMD virulence protein functions.

ASM does not own the copyrights to Supplemental Material that may be linked to, or accessed through, an article. The authors have granted ASM a non-exclusive, world-wide license to publish the Supplemental Material files. Please contact the corresponding author directly for reuse.

## References

[1] Daniels NA, MacKinnon L, Bishop R, Altekruse S, Ray B, Hammond RM, Thompson S, Wilson S, Bean NH, Griffin PM, Slutsker L. 2000. Vibrio parahaemolyticus infections in the United States, 1973-1998. J Infect Dis 181:1661–1666. doi:10.1086/315459 10823766

[2] Joseph SW, Colwell RR, Kaper JB. 1982*.* Vibrio parahaemolyticus and related Halophilic Vibrios. Crit Rev Microbiol 10:77–124. doi:10.3109/10408418209113506 6756788

[3] de Souza Santos M, Orth K. 2014. Intracellular Vibrio parahaemolyticus escapes the vacuole and establishes a replicative niche in the cytosol of epithelial cells. mBio 5:e01506–14. doi:10.1128/mBio.01506-14 25205094 PMC4173779

[4] McCarter L. 1999. The multiple identities of Vibrio parahaemolyticus. J Mol Microbiol Biotechnol 1:51–57. 10941784

[5] Billaud M, Seneca F, Tambutté E, Czerucka D. 2022. An increase of seawater temperature upregulates the expression of Vibrio parahaemolyticus virulence factors implicated in adhesion and biofilm formation. Front Microbiol 13:840628. doi:10.3389/fmicb.2022.840628 35350627 PMC8957992

[6] Nair GB, Ramamurthy T, Bhattacharya SK, Dutta B, Takeda Y, Sack DA. 2007. Global dissemination of Vibrio parahaemolyticus serotype O3:K6 and its Serovariants. Clin Microbiol Rev 20:39–48. doi:10.1128/CMR.00025-06 17223622 PMC1797631

[7] Boyd EF, Cohen ALV, Naughton LM, Ussery DW, Binnewies TT, Stine OC, Parent MA. 2008. Molecular analysis of the emergence of pandemic Vibrio parahaemolyticus. BMC Microbiol 8:110. doi:10.1186/1471-2180-8-110 18590559 PMC2491623

[8] Makino K, Oshima K, Kurokawa K, Yokoyama K, Uda T, Tagomori K, Iijima Y, Najima M, Nakano M, Yamashita A, Kubota Y, Kimura S, Yasunaga T, Honda T, Shinagawa H, Hattori M, Iida T. 2003. Genome sequence of Vibrio parahaemolyticus: a pathogenic mechanism distinct from that of V Cholerae. Lancet 361:743–749. doi:10.1016/S0140-6736(03)12659-1 12620739

[9] Hurley CC, Quirke A, Reen FJ, Boyd EF. 2006. Four genomic islands that mark post-1995 pandemic Vibrio parahaemolyticus isolates. BMC Genomics 7:104. doi:10.1186/1471-2164-7-104 16672049 PMC1464126

[10] Jana B, Keppel K, Fridman CM, Bosis E, Salomon D, Bordenstein S, Ramamurthy T. 2022. Multiple T6SSS, mobile auxiliary modules, and effectors revealed in a systematic analysis of the Vibrio parahaemolyticus pan-genome. mSystems 7. doi:10.1128/msystems.00723-22 PMC9765294 36226968

[11] Okuda J, Ishibashi M, Hayakawa E, Nishino T, Takeda Y, Mukhopadhyay AK, Garg S, Bhattacharya SK, Nair GB, Nishibuchi M. 1997. Emergence of a unique O3:K6 clone of Vibrio parahaemolyticus in Calcutta, India, and isolation of strains from the same clonal group from Southeast Asian travelers arriving in Japan. J Clin Microbiol 35:3150–3155. doi:10.1128/jcm.35.12.3150-3155.1997 9399511 PMC230139

[12] Reen FJ, Almagro-Moreno S, Ussery D, Boyd EF. 2006. The genomic code: inferring vibrionaceae niche specialization. Nat Rev Microbiol 4:697–704. doi:10.1038/nrmicro1476 16894340

[13] Ma L, Zhang Y, Yan X, Guo L, Wang L, Qiu J, Yang R, Zhou D. 2012. Expression of the type VI secretion system 1 component Hcp1 is indirectly repressed by Opar in Vibrio parahaemolyticus. ScientificWorldJournal 2012:982140. doi:10.1100/2012/982140 22924031 PMC3417189

[14] Izutsu K, Kurokawa K, Tashiro K, Kuhara S, Hayashi T, Honda T, Iida T. 2008. Comparative genomic analysis using microarray demonstrates a strong correlation between the presence of the 80-kilobase pathogenicity island and pathogenicity in Kanagawa phenomenon-positive Vibrio parahaemolyticus strains. Infect Immun 76:1016–1023. doi:10.1128/IAI.01535-07 18195030 PMC2258825

[15] Ceccarelli D, Hasan NA, Huq A, Colwell RR. 2013. Distribution and dynamics of epidemic and pandemic Vibrio parahaemolyticus virulence factors. Front Cell Infect Microbiol 3:97. doi:10.3389/fcimb.2013.00097 24377090 PMC3858888

[16] De Souza Santos M, Orth K. 2019. The role of the type III secretion system in the intracellular lifestyle of Enteric pathogens. Microbiol Spectr 7. doi:10.1128/microbiolspec.BAI-0008-2019 PMC11026088 31152523

[17] Salomon D, Gonzalez H, Updegraff BL, Orth K. 2013*.* Vibrio parahaemolyticus type VI secretion system 1 is activated in marine conditions to target bacteria, and is differentially regulated from system 2. PLoS One 8:e61086. doi:10.1371/journal.pone.0061086 23613791 PMC3628861

[18] Tettelin H, Masignani V, Cieslewicz MJ, Donati C, Medini D, Ward NL, Angiuoli SV, Crabtree J, Jones AL, Durkin AS, et al.. 2005. Genome analysis of multiple pathogenic isolates of Streptococcus agalactiae: implications for the microbial "pan-genome. Proc Natl Acad Sci U S A 102:13950–13955. doi:10.1073/pnas.0506758102 16172379 PMC1216834

[19] Kahlke T, Goesmann A, Hjerde E, Willassen NP, Haugen P. 2012. Unique core genomes of the bacterial family vibrionaceae: insights into niche adaptation and speciation. BMC Genomics 13:179. doi:10.1186/1471-2164-13-179 22574681 PMC3464603

[20] Hazen TH, Pan L, Gu JD, Sobecky PA. 2010. The contribution of mobile genetic elements to the evolution and ecology of vibrios. FEMS Microbiol Ecol 74:485–499. doi:10.1111/j.1574-6941.2010.00937.x 20662928

[21] Carr VR, Shkoporov A, Hill C, Mullany P, Moyes DL. 2021. Probing the mobilome: discoveries in the dynamic microbiome. Trends Microbiol 29:158–170. doi:10.1016/j.tim.2020.05.003 32448763

[22] Kim Y, Gu C, Kim HU, Lee SY. 2020. Current status of pan-genome analysis for pathogenic bacteria. Curr Opin Biotechnol 63:54–62. doi:10.1016/j.copbio.2019.12.001 31891864

[23] Shapiro BJ. 2017. The population genetics of pangenomes. Nat Microbiol 2:1574. doi:10.1038/s41564-017-0066-6 29176697

[24] McInerney JO, McNally A, O’Connell MJ. 2017. Reply to 'the population genetics of pangenomes Nat Microbiol 2:1575. doi:10.1038/s41564-017-0068-4 29176701

[25] Sapegin DI, Baĭrammuradova MK. 1988. Hygienic assessment of water for simultaneous determination of magnesium chlorate and ammonium nitrate. Gig Sanit, no. 2:9–11. 2836272

[26] Dunbrack RL. 2006. Sequence comparison and protein structure prediction. Curr Opin Struct Biol 16:374–384. doi:10.1016/j.sbi.2006.05.006 16713709

[27] Moult J. 2005. A decade of CASP: progress, bottlenecks and prognosis in protein structure prediction. Curr Opin Struct Biol 15:285–289. doi:10.1016/j.sbi.2005.05.011 15939584

[28] Pearson WR, Sierk ML. 2005. The limits of protein sequence comparison Curr Opin Struct Biol 15:254–260. doi:10.1016/j.sbi.2005.05.005 15919194 PMC2845305

[29] Valas RE, Yang S, Bourne PE. 2009. Nothing about protein structure classification makes sense except in the light of evolution. Curr Opin Struct Biol 19:329–334. doi:10.1016/j.sbi.2009.03.011 19394812 PMC2696554

[30] Choi IG, Kim SH. 2006. Evolution of protein structural classes and protein sequence families. Proc Natl Acad Sci U S A 103:14056–14061. doi:10.1073/pnas.0606239103 16959887 PMC1560931

[31] Aravind L, Mazumder R, Vasudevan S, Koonin EV. 2002. Trends in protein evolution inferred from sequence and structure analysis. Curr Opin Struct Biol 12:392–399. doi:10.1016/s0959-440x(02)00334-2 12127460

[32] Li P, Rivera-Cancel G, Kinch LN, Salomon D, Tomchick DR, Grishin NV, Orth K. 2016. Bile salt receptor complex activates a pathogenic type III secretion system. Elife 5:e15718. doi:10.7554/eLife.15718 27377244 PMC4933562

[33] Cheng H, Schaeffer RD, Liao Y, Kinch LN, Pei J, Shi S, Kim B-H, Grishin NV. 2014. ECOD: an evolutionary classification of protein domains. PLoS Comput Biol 10:e1003926. doi:10.1371/journal.pcbi.1003926 25474468 PMC4256011

[34] Mak H, Thurston TLM. 2021. Interesting biochemistries in the structure and function of bacterial effectors. Front Cell Infect Microbiol 11:608860. doi:10.3389/fcimb.2021.608860 33718265 PMC7943720

[35] Kinch LN, Schaeffer RD, Kryshtafovych A, Grishin NV. 2021. Target classification in the 14th round of the critical assessment of protein structure prediction (CASP14). Proteins 89:1618–1632. doi:10.1002/prot.26202 34350630 PMC8616802

[36] Baek M, DiMaio F, Anishchenko I, Dauparas J, Ovchinnikov S, Lee GR, Wang J, Cong Q, Kinch LN, Schaeffer RD, et al.. 2021. Accurate prediction of protein structures and interactions using a three-track neural network. Science 373:871–876. doi:10.1126/science.abj8754 34282049 PMC7612213

[37] Jumper J, Evans R, Pritzel A, Green T, Figurnov M, Ronneberger O, Tunyasuvunakool K, Bates R, Žídek A, Potapenko A, et al.. 2021. Highly accurate protein structure prediction with AlphaFold. Nature 596:583–589. doi:10.1038/s41586-021-03819-2 34265844 PMC8371605

[38] Kinch LN, Pei J, Kryshtafovych A, Schaeffer RD, Grishin NV. 2021. Topology evaluation of models for difficult targets in the 14th round of the critical assessment of protein structure prediction. Proteins 89:1673–1686. doi:10.1002/prot.26172 34240477 PMC8616777

[39] Jumper J, Evans R, Pritzel A, Green T, Figurnov M, Ronneberger O, Tunyasuvunakool K, Bates R, Žídek A, Potapenko A, et al.. 2021. Applying and improving AlphaFold at CASP14. Proteins 89:1711–1721. doi:10.1002/prot.26257 34599769 PMC9299164

[40] Anishchenko I, Baek M, Park H, Hiranuma N, Kim DE, Dauparas J, Mansoor S, Humphreys IR, Baker D. 2021. Protein tertiary structure prediction and refinement using deep learning and rosetta in CASP14. Proteins 89:1722–1733. doi:10.1002/prot.26194 34331359 PMC8616808

[41] Kinch LN, Cong Q, Jaishankar J, Orth K. 2022. Co-component signal transduction systems: fast-evolving virulence regulation cassettes discovered in enteric bacteria. Proc Natl Acad Sci U S A 119:e2203176119. doi:10.1073/pnas.2203176119 35648808 PMC9214523

[42] Varadi M, Anyango S, Deshpande M, Nair S, Natassia C, Yordanova G, Yuan D, Stroe O, Wood G, Laydon A, et al.. 2022. AlphaFold protein structure database: massively expanding the structural coverage of protein-sequence space with high-accuracy models. Nucleic Acids Res 50:D439–D444. doi:10.1093/nar/gkab1061 34791371 PMC8728224

[43] Zhang J, Schaeffer RD, Durham J, Cong Q, Grishin NV. 2022. DPAM: a domain parser for AlphaFold models. Bioinformatics. doi:10.1101/2022.09.22.509116 PMC9850437 36539305

[44] Schaeffer RD, Zhang J, Kinch LN, Pei J, Cong Q, Grishin NV. 2023. Classification of domains in predicted structures of the human Proteome. Proc Natl Acad Sci U S A 120:e2214069120. doi:10.1073/pnas.2214069120 36917664 PMC10041065

[45] Cheng H, Liao Y, Schaeffer RD, Grishin NV. 2015. Manual classification strategies in the ECOD database. Proteins 83:1238–1251. doi:10.1002/prot.24818 25917548 PMC4624060

[46] Medvedev KE, Kinch LN, Dustin Schaeffer R, Pei J, Grishin NV. 2021. A fifth of the protein world: rossmann-like proteins as an evolutionarily successful structural unit. J Mol Biol 433:166788. doi:10.1016/j.jmb.2020.166788 33387532 PMC7870570

[47] Kozma D, Simon I, Tusnády GE. 2013. PDBTM: protein data bank of transmembrane proteins after 8 years. Nucleic Acids Res 41:D524–9. doi:10.1093/nar/gks1169 23203988 PMC3531219

[48] Jeckelmann JM, Erni B. 2020. Transporters of glucose and other carbohydrates in bacteria. Pflugers Arch 472:1129–1153. doi:10.1007/s00424-020-02379-0 32372286

[49] Sonnhammer EL, Eddy SR, Durbin R. 1997. Pfam: a comprehensive database of protein domain families based on seed alignments. Proteins 28:405–420. doi:10.1002/(sici)1097-0134(199707)28:3<405::aid-prot10>3.0.co;2-l 9223186

[50] Flower DR. 1996. The lipocalin protein family: structure and function. Biochem J 318 (Pt 1):1–14. doi:10.1042/bj3180001 8761444 PMC1217580

[51] Cortés-Avalos D, Martínez-Pérez N, Ortiz-Moncada MA, Juárez-González A, Baños-Vargas AA, Estrada-de Los Santos P, Pérez-Rueda E, Ibarra JA. 2021. An update of the unceasingly growing and diverse AraC/XylS family of transcriptional activators. FEMS Microbiol Rev 45:fuab020. doi:10.1093/femsre/fuab020 33837749

[52] Fridman CM, Keppel K, Gerlic M, Bosis E, Salomon D. 2020. A comparative genomics methodology reveals a widespread family of membrane-disrupting T6SS effectors. Nat Commun 11:1085. doi:10.1038/s41467-020-14951-4 32109231 PMC7046647

[53] Jensen RV, Depasquale SM, Harbolick EA, Hong T, Kernell AL, Kruchko DH, Modise T, Smith CE, McCarter LL, Stevens AM. 2013. Complete genome sequence of prepandemic Vibrio parahaemolyticus BB22OP. Genome Announc 1:e00002-12. doi:10.1128/genomeA.00002-12 PMC3587917 23469330

[54] Chen Y, Stine OC, Badger JH, Gil AI, Nair GB, Nishibuchi M, Fouts DE. 2011. Comparative genomic analysis of Vibrio parahaemolyticus: Serotype conversion and virulence. BMC Genomics 12:294. doi:10.1186/1471-2164-12-294 21645368 PMC3130711

[55] Bian S, Zeng W, Li Q, Li Y, Wong N-K, Jiang M, Zuo L, Hu Q, Li L. 2020. Genetic structure, function, and evolution of capsule biosynthesis loci in Vibrio parahaemolyticus Front Microbiol 11:546150. doi:10.3389/fmicb.2020.546150 33505361 PMC7829505

[56] Borodovich T, Shkoporov AN, Ross RP, Hill C. 2022. Phage-mediated horizontal gene transfer and its implications for the human gut Microbiome. Gastroenterol Rep (Oxf) 10:goac012. doi:10.1093/gastro/goac012 35425613 PMC9006064

[57] Labrie SJ, Samson JE, Moineau S. 2010. Bacteriophage resistance mechanisms. Nat Rev Microbiol 8:317–327. doi:10.1038/nrmicro2315 20348932

[58] Bari SMN, Chou-Zheng L, Howell O, Hossain M, Hill CM, Boyle TA, Cater K, Dandu VS, Thomas A, Aslan B, Hatoum-Aslan A. 2022. A unique mode of nucleic acid immunity performed by a multifunctional bacterial enzyme. Cell Host Microbe 30:570–582. doi:10.1016/j.chom.2022.03.001 35421352

[59] Xu F, Gonzalez-Escalona N, Drees KP, Sebra RP, Cooper VS, Jones SH, Whistler CA. 2017. Parallel evolution of two clades of an atlantic-endemic pathogenic lineage of Vibrio parahaemolyticus by independent acquisition of related pathogenicity islands. Appl Environ Microbiol 83:e01168-17. doi:10.1128/AEM.01168-17 28687650 PMC5583489

[60] Liu Y, Zeng H, Hou Y, Li Z, Li L, Song X, Ding J, Shao F, Xu Y, Heran Darwin K. 2022. Calmodulin binding activates chromobacterium CopC effector to ADP-riboxanate host apoptotic caspases . mBio 13:e0069022. doi:10.1128/mbio.00690-22 35446120 PMC9239266

[61] Ploy MC, Lambert T, Couty JP, Denis F. 2000. Integrons: an antibiotic resistance gene capture and expression system. Clin Chem Lab Med 38:483–487. doi:10.1515/CCLM.2000.070 10987194

[62] Wen Y, Behiels E, Felix J, Elegheert J, Vergauwen B, Devreese B, Savvides SN. 2014. The bacterial Antitoxin HipB establishes a ternary complex with operator DNA and phosphorylated toxin HipA to regulate bacterial persistence. Nucleic Acids Res 42:10134–10147. doi:10.1093/nar/gku665 25056321 PMC4150777

[63] Nirwan N, Itoh Y, Singh P, Bandyopadhyay S, Vinothkumar KR, Amunts A, Saikrishnan K. 2019. Structure-based mechanism for activation of the AAA+ Gtpase McrB by the endonuclease McrC. Nat Commun 10:3058. doi:10.1038/s41467-019-11084-1 31296862 PMC6624300

[64] Broberg CA, Zhang L, Gonzalez H, Laskowski-Arce MA, Orth K. 2010. A Vibrio effector protein is an inositol phosphatase and disrupts host cell membrane integrity. Science 329:1660–1662. doi:10.1126/science.1192850 20724587

[65] Cheng Y-S, Shi Z, Doudeva LG, Yang W-Z, Chak K-F, Yuan HS. 2006. High-resolution crystal structure of a truncated cole7 translocation domain: implications for colicin transport across membranes. J Mol Biol 356:22–31. doi:10.1016/j.jmb.2005.11.056 16360169

[66] Tchelet D, Keppel K, Bosis E, Salomon D. 2023. Vibrio parahaemolyticus T6SS2 effector repertoires. Gut Microbes 15:2178795. doi:10.1080/19490976.2023.2178795 36803660 PMC9980498

[67] Ridley CP, Lee HY, Khosla C. 2008. Evolution of polyketide synthases in bacteria. Proc Natl Acad Sci U S A 105:4595–4600. doi:10.1073/pnas.0710107105 18250311 PMC2290765

[68] Li Z, Liu W, Fu J, Cheng S, Xu Y, Wang Z, Liu X, Shi X, Liu Y, Qi X, Liu X, Ding J, Shao F. 2021. Shigella evades pyroptosis by arginine ADP-riboxanation of caspase-11. Nature 599:290–295. doi:10.1038/s41586-021-04020-1 34671164

[69] Gonzalez R, Murarka A, Dharmadi Y, Yazdani SS. 2008. A new model for the anaerobic fermentation of glycerol in enteric bacteria: trunk and auxiliary pathways in Escherichia coli. Metab Eng 10:234–245. doi:10.1016/j.ymben.2008.05.001 18632294

[70] Fan F, Li Z, Wang J, Diao B, Liang W, Kan B. 2021. A PolyQ membrane protein of Vibrio cholerae acts as the receptor for phage infection. J Virol 95:e02245-20. doi:10.1128/JVI.02245-20 33408174 PMC8094955

[71] Dar Y, Jana B, Bosis E, Salomon D. 2022. A binary effector module secreted by a type VI secretion system. EMBO Rep 23:e53981. doi:10.15252/embr.202153981 34752000 PMC8728615

[72] Salomon D, Kinch LN, Trudgian DC, Guo X, Klimko JA, Grishin NV, Mirzaei H, Orth K. 2014. Marker for type VI secretion system effectors. Proc Natl Acad Sci U S A 111:9271–9276. doi:10.1073/pnas.1406110111 24927539 PMC4078801

[73] Craig L, Taylor RK, Pique ME, Adair BD, Arvai AS, Singh M, Lloyd SJ, Shin DS, Getzoff ED, Yeager M, Forest KT, Tainer JA. 2003. Type IV pilin structure and assembly: X-ray and EM analyses of Vibrio cholerae toxin-coregulated pilus and Pseudomonas aeruginosa PAK pilin. Mol Cell 11:1139–1150. doi:10.1016/s1097-2765(03)00170-9 12769840

[74] O’Boyle N, Houeix B, Kilcoyne M, Joshi L, Boyd A. 2013. The MSHA pilus of Vibrio parahaemolyticus has lectin functionality and enables TTSS-mediated pathogenicity. Int J Med Microbiol 303:563–573. doi:10.1016/j.ijmm.2013.07.010 23981476

[75] Frischkorn KR, Stojanovski A, Paranjpye R. 2013. Vibrio parahaemolyticus type IV pili mediate interactions with diatom-derived chitin and point to an unexplored mechanism of environmental persistence. Environ Microbiol 15:1416–1427. doi:10.1111/1462-2920.12093 23441888

[76] Tocilj A, Munger C, Proteau A, Morona R, Purins L, Ajamian E, Wagner J, Papadopoulos M, Van Den Bosch L, Rubinstein JL, Féthière J, Matte A, Cygler M. 2008. Bacterial polysaccharide co-polymerases share a common framework for control of polymer length. Nat Struct Mol Biol 15:130–138. doi:10.1038/nsmb.1374 18204465

[77] Sathiyamoorthy K, Mills E, Franzmann TM, Rosenshine I, Saper MA. 2011. The crystal structure of Escherichia coli group 4 capsule protein GfcC reveals a domain organization resembling that of Wza. Biochemistry 50:5465–5476. doi:10.1021/bi101869h 21449614

[78] Holm L. 2019. Benchmarking fold detection by Dalilite V.5. Bioinformatics 35:5326–5327. doi:10.1093/bioinformatics/btz536 31263867

[79] Steinegger M, Meier M, Mirdita M, Vöhringer H, Haunsberger SJ, Söding J. 2019. HH-suite3 for fast remote Homology detection and deep protein annotation. BMC Bioinformatics 20:473. doi:10.1186/s12859-019-3019-7 31521110 PMC6744700

[80] Kabsch W, Sander C. 1983. Dictionary of protein secondary structure: pattern recognition of hydrogen-bonded and geometrical features. Biopolymers 22:2577–2637. doi:10.1002/bip.360221211 6667333

[81] Avram O, Rapoport D, Portugez S, Pupko T. 2019. M1CR0B1AL1Z3R-a user-friendly web server for the analysis of large-scale microbial genomics data. Nucleic Acids Res 47:W88–W92. doi:10.1093/nar/gkz423 31114912 PMC6602433

[82] Li W, O’Neill KR, Haft DH, DiCuccio M, Chetvernin V, Badretdin A, Coulouris G, Chitsaz F, Derbyshire MK, Durkin AS, Gonzales NR, Gwadz M, Lanczycki CJ, Song JS, Thanki N, Wang J, Yamashita RA, Yang M, Zheng C, Marchler-Bauer A, Thibaud-Nissen F. 2021. RefSeq: expanding the prokaryotic genome annotation pipeline reach with protein family model curation. Nucleic Acids Res 49:D1020–D1028. doi:10.1093/nar/gkaa1105 33270901 PMC7779008

[83] Brettin T, Davis JJ, Disz T, Edwards RA, Gerdes S, Olsen GJ, Olson R, Overbeek R, Parrello B, Pusch GD, Shukla M, Thomason JA 3rd, Stevens R, Vonstein V, Wattam AR, Xia F. 2015. RASTtk: a modular and extensible implementation of the RAST algorithm for building custom annotation pipelines and annotating batches of genomes. Sci Rep 5:8365. doi:10.1038/srep08365 25666585 PMC4322359

[84] Overbeek R, Begley T, Butler RM, Choudhuri JV, Chuang H-Y, Cohoon M, de Crécy-Lagard V, Diaz N, Disz T, Edwards R, et al.. 2005. The subsystems approach to genome annotation and its use in the project to annotate 1000 Genomes. Nucleic Acids Res 33:5691–5702. doi:10.1093/nar/gki866 16214803 PMC1251668

